# Supplementary material for: A systematic analysis of atomic protein–ligand interactions in the PDB
Source: Medchemcomm. 2017 Sep 26;8(10):1970–81. doi: 10.1039/c7md00381a (PMC5708362; doi:10.1039/c7md00381a)
Supplement: Supplementary file 1 [file MD-008-C7MD00381A-s001.pdf]

Electronic Supplementary Material (ESI) for MedChemComm.
This journal is © The Royal Society of Chemistry 2017

SUPPORT INFORMATION

A Systematic Analysis of Atomic Protein-Ligand Interactions from the PDB

Renato Ferreira de Freitas[1], Matthieu Schapira[1,2,*]

[1] Structural Genomics Consortium, University of Toronto, Toronto, ON M5G 1L7, Canada

[2] Department of Pharmacology and Toxicology, University of Toronto, Toronto, ON M5S 1A8, Canada

p.1 Title Page

p2. Computational Methods

p3. Figure S1. Distance and angles used to search for non-covalent interactions involving aromatic rings; a) π-stacking, b) amide stacking. The angle θ is the planar angle between two rings or one ring and the amide bond

p4. Figure S2. Distance and angles used to search for halogen bonding (a) and multipolar halogen interaction (b).

p6. Table S2. Most frequent protein-ligand interactions extracted from PDB.

p8. Figure S3. Selected molecular properties for the 11016 ligands in the database

p8. Figure S4. Distribution of the occurrence of the hydrophobic (aliphatic-aromatic) interactions with each amino acid.

p9 Figure S5. 3D scatter plot of the hydrophobic interactions involving and aromatic carbon from the receptor and an aliphatic carbon from the ligand.

p9. Figure S6. Distribution of the occurrence of the N-H…O interactions with each amino acid.

p10. Figure S7. Distribution of the occurrence of the O-H…O interactions with each amino acid.

p10. Figure S8. (a) Distribution of the occurrence of the π-π stacking interactions with each amino acid.

p11. Figure S9. (a) Distribution of the occurrence of the C-H…O interactions with each amino acid.

p12. Figure S10. (a) Distribution of the occurrence of the salt bridge interactions with each amino acid; (b) 3D scatter plot of the salt bridge interactions.
p12. Figure S11. Distribution of the occurrence of the amide-π stacking interactions with each amino acid.
p13. Figure S12. Distribution of the occurrence of the cation-π stacking interactions with each amino acid.
p13. Figure S13. Distribution of the occurrence of the halogen bonding interactions with each amino acid.
p14. Figure S14. Distribution of the occurrence of the halogen multipolar interactions with each amino acid.

Computational Methods

In this study, we downloaded all protein-ligand complexes available in the PDB[1] (76056 PDB entries). They were extracted from a snapshot of PDB captured on September 25, 2016, and filtered using the following criteria: (i) crystal structures with resolution ≤ 2.5 Å, (ii) included at least one small ligand with a heavy atom count ≥6 and ≤100, (iii) structures classified as DNA or RNA were eliminated, (iv) structures that do not contain a protein molecule, but only contain a DNA or RNA molecule complexed to a ligand were excluded, (v) we removed all ligands containing phosphorous in their structures (including nucleotides and nucleotide-like analogues) (vi) compounds of low interest for medicinal chemistry applications (such as buffers) were removed using the BioLiP list.[2] These selection criteria led to a set of 11,016 PDB entries. All the complexes were converted into ICM objects using Molsoft ICM suite.[3] In this step, alternative conformations/models molecules were removed, hydrogens were added and energy-minimized, water hydrogen rotameric state and protonation states were fixed, missing side chains in the protein were inserted, and partial charges and atom types were assigned.

We wrote an icm script to examine the environment of proteins-ligand contacts. In the first step, the script identifies all ligand and protein atoms that come within 4.0 Å

of each other. These close-contact atoms are then characterized according to their respective icm and mmff atom types. In the second step, for each ligand-protein atom pair, the respective ligand and protein atoms are expanded to include the atoms in question and their successive neighbors. In addition, the script also extracts the distance between the interacting atoms, their coordinates, as well as the coordinates for the neighboring atoms. Finally, each ligand-protein atom pair that had the same icm atom types were grouped together and saved as icm tables for further analysis.

Hydrophobic contacts were identified when a ligand carbon (or halogen) atom comes within 4.0 Å of a receptor carbon (or sulfur) atom. A hydrogen bond was identified if the hydrogen-bond donor was within 3.9 Å of the hydrogen-bond acceptor, and the angle formed between the donor, the hydrogen atom, and the acceptor, θ, was larger than 90°.[4] To account for the hydrogen bonds mediated by water between the protein and the ligand, the water were considered as part of the receptor and only the ones tightly bound (2-3 hydrogen bond coordination) were kept for analysis.

For weak hydrogen bonding, the distance and angle cutoffs were 3.6 Å and 130°, respectively.[4] Potential salt bridges were detected when two atoms of opposite charges were in a contact distance below 4.0 Å. The same cutoff distance was applied to a positively charged nitrogen close to an aromatic carbon to identify potential cation-π interactions. For interactions involving two aromatic rings (or an amide and a ring), the planar angle was calculated (Figure S1) as well as the angle between the two vectors normal to the planes (θ). An edge-to-face π- π stacking interaction was detected if the angle θ was between 60° and 120° (60° ≤ θ □≤ 120°). If $\theta \leq 30°$ or ≥ 150° the interaction was considered as a face-to-face π- π stacking interaction. The same criteria were applied to the amide stacking interactions.

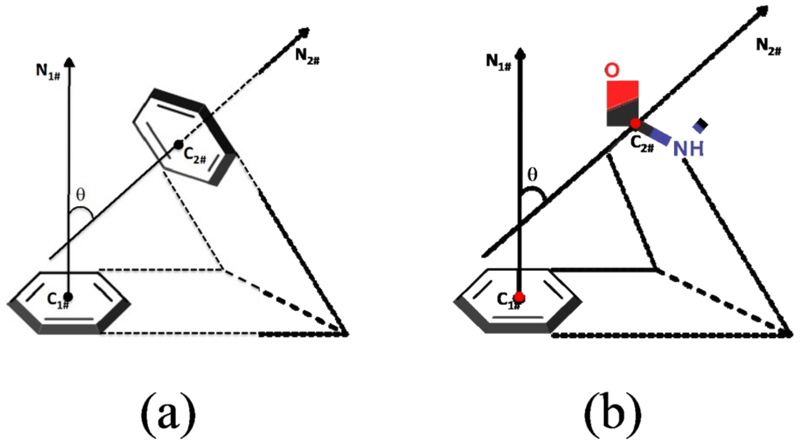

Figure S1. Distance and angles used to search for non-covalent interactions involving aromatic rings; a) π-stacking, b) amide stacking. The angle θ is the planar angle between two rings or one ring and the amide bond.

For halogen bonding interactions we collected only those with X···A distances that are less than their respective van der Walls radius sums plus 0.2 Å to account for the uncertainties in the distances (Figure S2a and Table S1 for the cutoff values) as well as with halogen bonding angles 130° ≤ α1 ≤ 180° 90° ≤ α2 ≤ 150°. For multipolar halogen interactions, we also only considered the contacts that are less than their respective van der Walls radius sums plus 0.2 Å and the angles Θ1 ≥ 140° and 70° ≤ Θ2 ≤ 110° (Figure S2b).

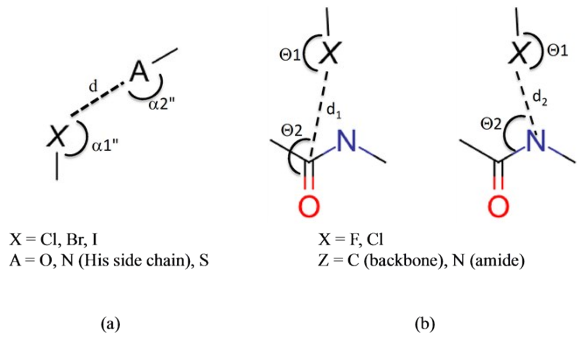

Figure S2. Distance and angles used to search for halogen bonding (a) and multipolar halogen interaction (b).

We used the fit quality (FQ) score to measure ligand efficiency of ligands with different sizes (10.1021/jm701255b)[5]

$$FQ = LE/LE_{scale}$$

$$LE_{scale} = 0.0715+7.5328/(HA)+25.7079/(HA^2)-361.4722/(HA^3)$$

References

(1) Berman, H. M. The Protein Data Bank. *Nucleic Acids Res.* **2000**, *28*, 235–242.

(2) Yang, J.; Roy, A.; Zhang, Y. BioLiP: A Semi-Manually Curated Database for Biologically Relevant Ligand-Protein Interactions. *Nucleic Acids Res.* **2013**, *41*, D1096-103.

(3) Abagyan, R.; Totrov, M. Biased Probability Monte Carlo Conformational Searches and Electrostatic Calculations for Peptides and Proteins. *J. Mol. Biol.* **1994**, *235*, 983–1002.

(4) Jubb, H. C.; Higueruelo, A. P.; Ochoa-Montaño, B.; Pitt, W. R.; Ascher, D. B.; Blundell, T. L. Arpeggio: A Web Server for Calculating and Visualising Interatomic Interactions in Protein Structures. *J. Mol. Biol.* **2016**, *429*, 365–371.

(5) Reynolds, C. H.; Tounge, B. A.; Bembenek, S. D. Ligand Binding Efficiency: Trends, Physical Basis, and Implications. *J. Med. Chem.* **2008**, *51*, 2432–2438.

Table S2. Most frequent protein-ligand interactions extracted from PDB

Interaction	Protein Atom	Ligand Atom	Distance (Å)	Angle (°)	# Interactions
Hydrophobic					
aliphatic-aromatic	C ali	C aro	≤ 4.0		42443
aromatic-aliphatic	C aro	C ali	≤ 4.0		8899
aliphatic-aliphatic	C ali	C ali	≤ 4.0		8974
carbon-halogen	C	X	≤ 4.0		5147
sulfur-aromatic	S	C aro	≤ 4.0		1309
Hydrogen bonding					
O-H•••O	O neg	OH	≤ 3.9	≥ 90	4959
O-H•••O	OH	O neg	≤ 3.9	≥ 90	1221
O-H•••O	O	OH	≤ 3.9	≥ 90	1296
O-H•••O	OH	O	≤ 3.9	≥ 90	775
N-H•••O	NH	O	≤ 3.9	≥ 90	4872
N-H•••O	O	NH	≤ 3.9	≥ 90	2682
N-H•••O	NH	O neg	≤ 3.9	≥ 90	1441
N-H•••O	O neg	NH	≤ 3.9	≥ 90	1565
N-H•••O	NH pos	O	≤ 3.9	≥ 90	2904
N-H•••O	O	NH pos	≤ 3.9	≥ 90	1641
N-H•••N	NH	N	≤ 3.9	≥ 90	333
O•••WAT	Owat	O	≤ 3.9	≥ 90	3131
N•••WAT	Owat	N	≤ 3.9	≥ 90	1757
□-Stacking					
π-stacking (edge to face)	C aro	C aro	≤ 4.0	60 ≤ θ ≤ 120	8704
π-stacking (face to face)	C aro	C aro	≤ 4.0	≤ 30 or ≥ 150	8537
Weak hydrogen bonding					
C-H•••O	O	CH aro	≤ 3.6	≥ 130	4927
C-H•••O	CH aro	O	≤ 3.6	≥ 130	702
C-H•••O	CH ali	O	≤ 3.6	≥ 130	5316
C-H•••O	O	CH ali	≤ 3.6	≥ 130	2655
Salt bridge					
N•••O	N pos	O neg	≤ 4.0		4882
O•••N	O neg	N pos	≤ 4.0		2394
Amide stacking					
Amide stacking (face to face)	C (amide)	C aro	≤ 4.0	≤ 30 or ≥ 150	2907

Amide stacking (edge to face)	C (amide)	C aro	≤ 4.0	60 ≤ θ ≤ 120	2060
Cation-π					
N pos•••C aro	N pos	C aro	≤ 4.0		2338
N pos•••C aro	C aro	N pos	≤ 4.0		239
Halogen bonding					
Cl•••O	O	Cl	≤3.47	130° ≤ α1 ≤ 180°<br>90° ≤ α2 ≤ 150°	203
Cl•••N	N	Cl	≤3.5		1
Cl•••S	S	Cl	≤3.75		18
Br•••O	O	Br	≤3.57	130° ≤ α1 ≤ 180°<br>90° ≤ α2 ≤ 150°	86
Br•••N	N	Br	≤3.6		1
Br•••S	S	Br	≤3.85		4
I•••O	O	I	≤3.7	130° ≤ α1 ≤ 180°<br>90° ≤ α2 ≤ 150°	33
I•••N	N	I	≤3.73		0
I•••S	S	I	≤3.98		5
Multipolar halogen interaction					
F•••C	C (amide)	F	≤ 3.37	Θ1 ≥ 140°<br>70° ≤ Θ2 ≤ 110°	61
F•••N	N (amide)	F	≤ 3.22		48
Cl•••C	C (amide)	Cl	≤ 3.65	Θ1 ≥ 140°<br>70° ≤ Θ2 ≤ 110°	37
Cl•••N	N (amide)	Cl	≤ 3.50		28

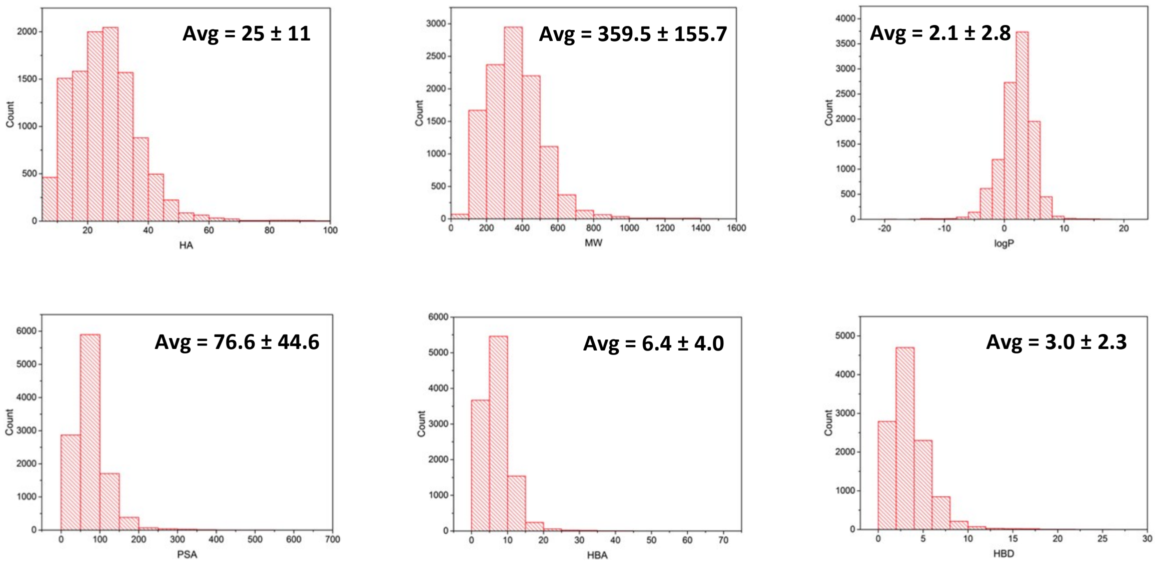

Figure S3. Selected molecular properties for the 11016 ligands in the database.

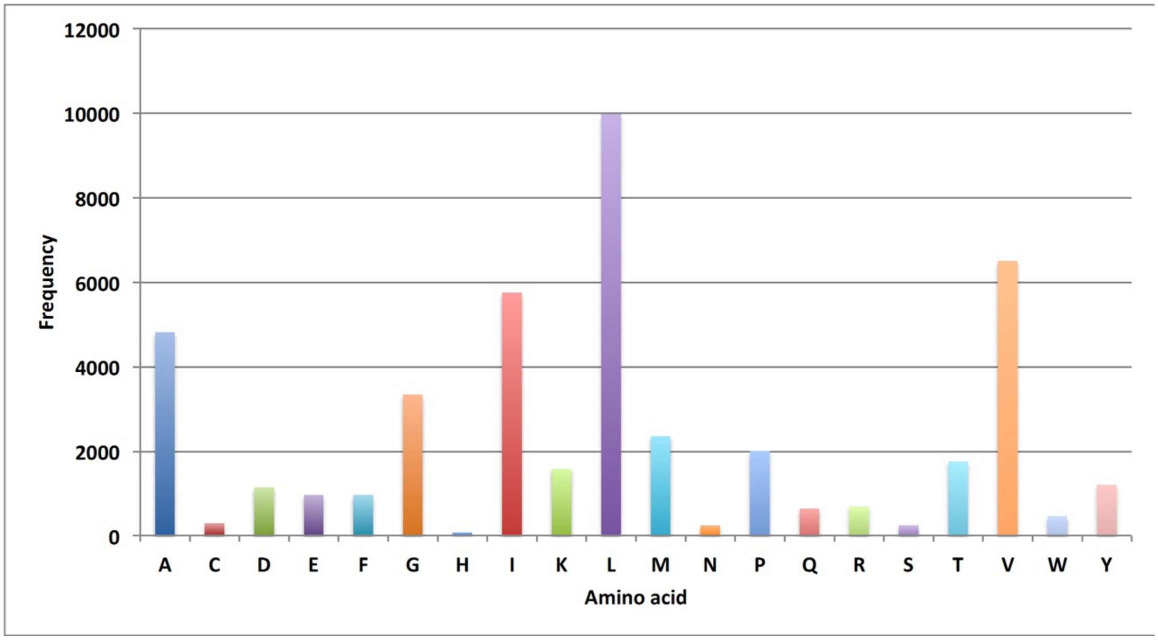

Figure S4. Frequency distribution of hydrophobic (aliphatic-aromatic) interactions with each amino acid.

.

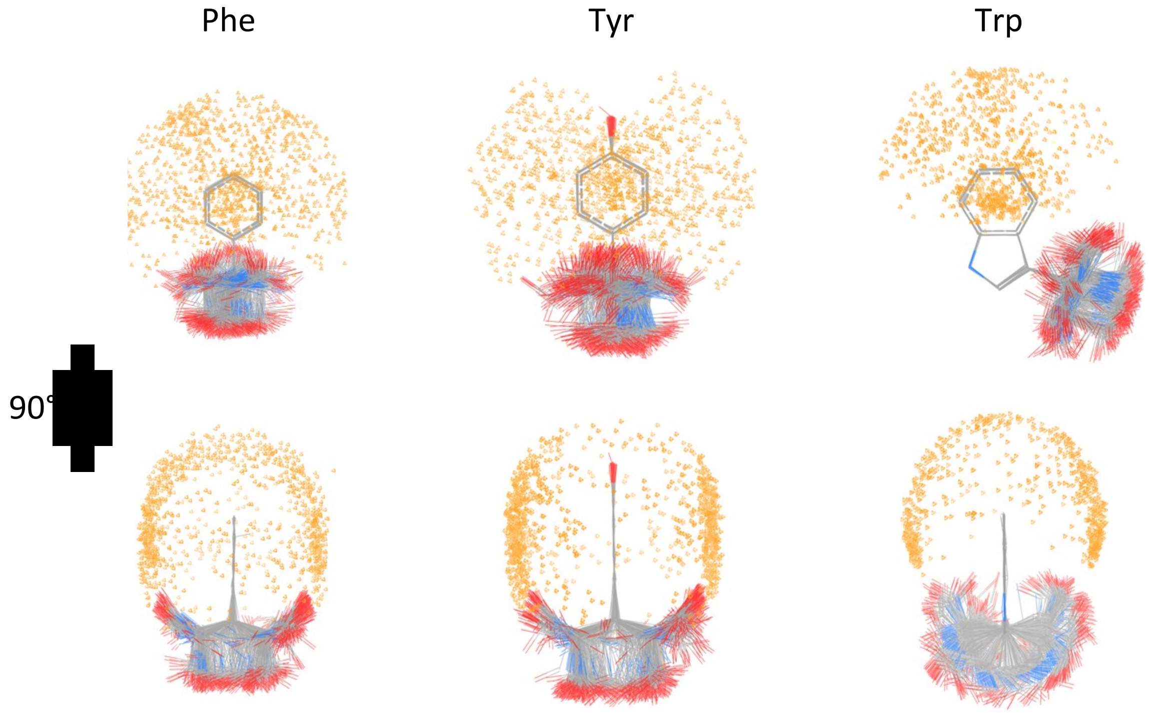

Figure S5. 3D scatter plot of the hydrophobic interactions involving an aromatic carbon from the receptor and an aliphatic carbon from the ligand.

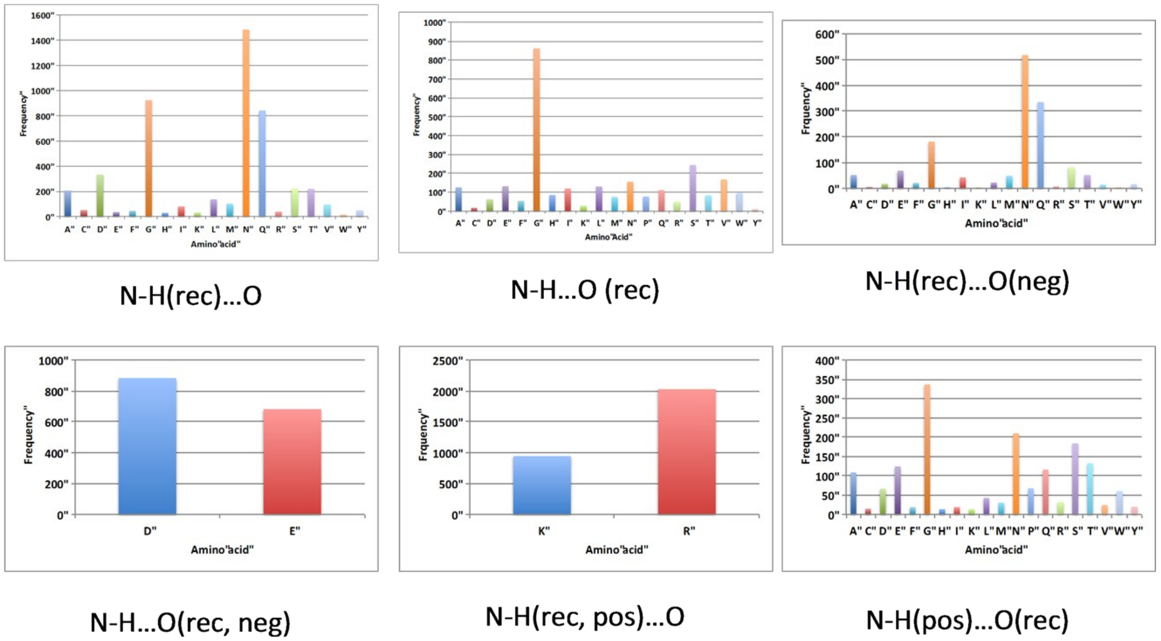

Figure S6. Frequency distribution of N-H…O interactions with each amino acid.

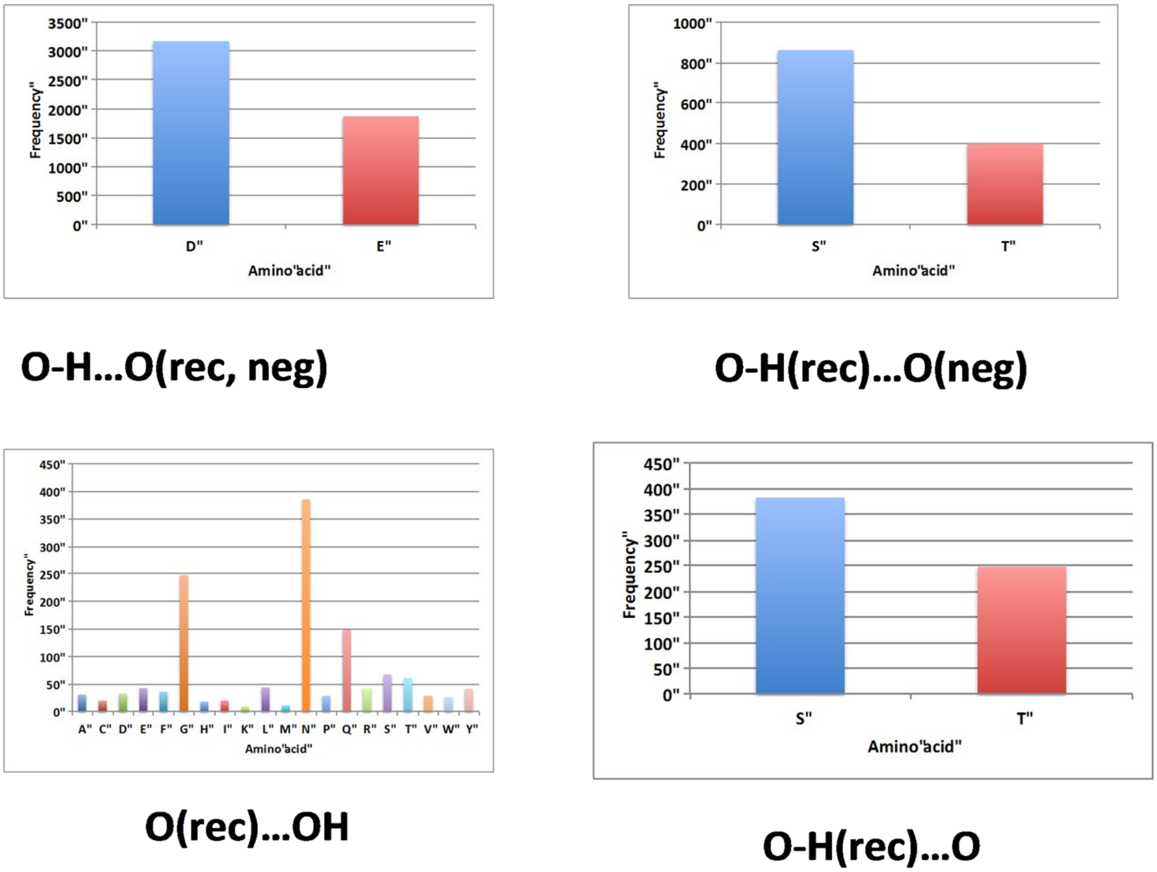

Figure S7. Distribution of the occurrence of the O-H…O interactions with each amino acid.

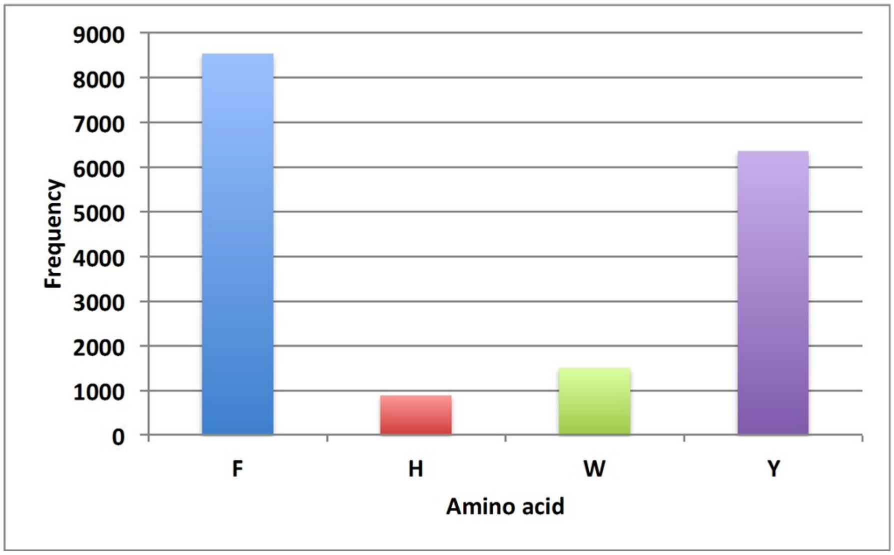

Figure S8. (a) Distribution of the occurrence of the π-π stacking interactions with each amino acid.

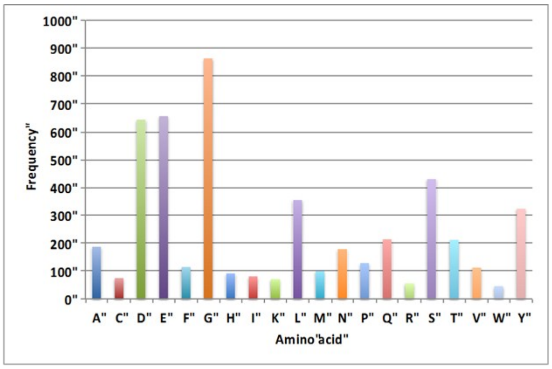

C-H (aro)…O (rec)

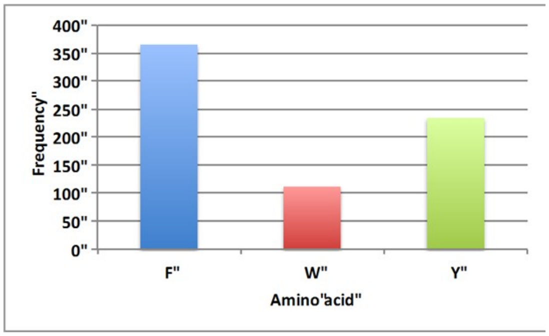

C-H (aro/rec)…O

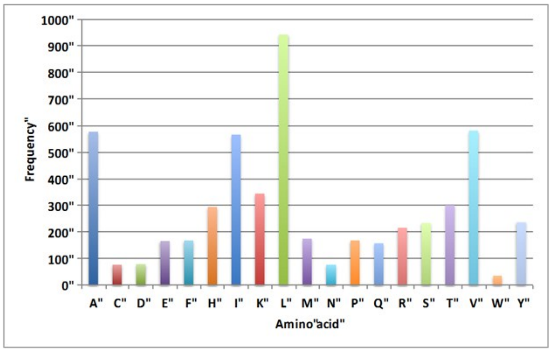

C-H (rec)…O

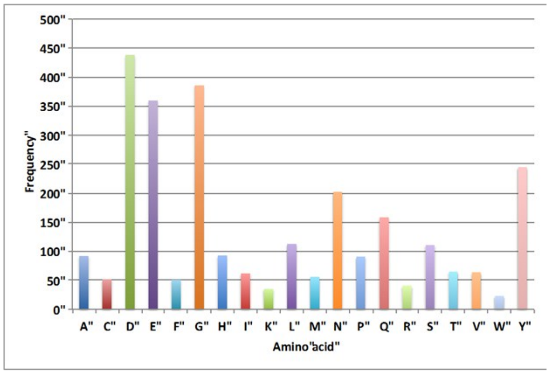

C-H (ali)…O(rec)

Figure S9. Distribution of the occurrence of the C-H…O interactions with each amino acid

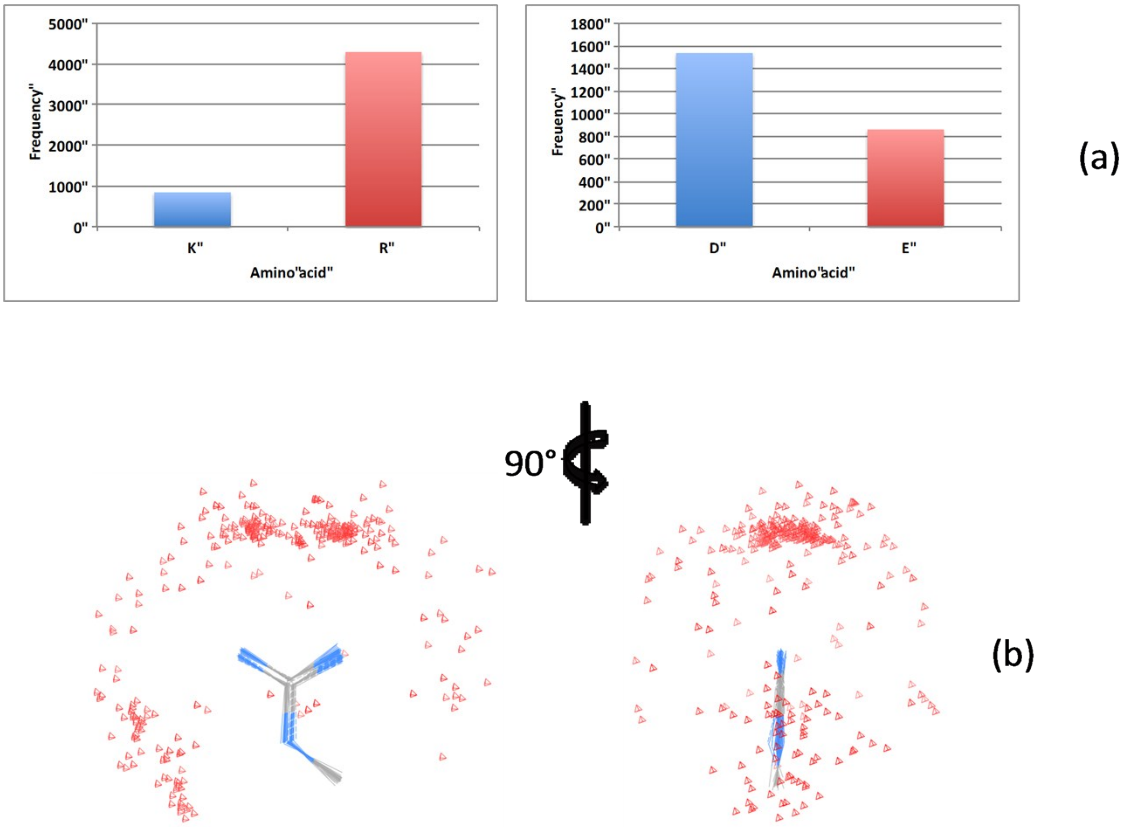

Figure S10. (a) Distribution of the occurrence of the salt bridge interactions with each amino acid; (b) 3D scatter plot of the salt bridge interactions.

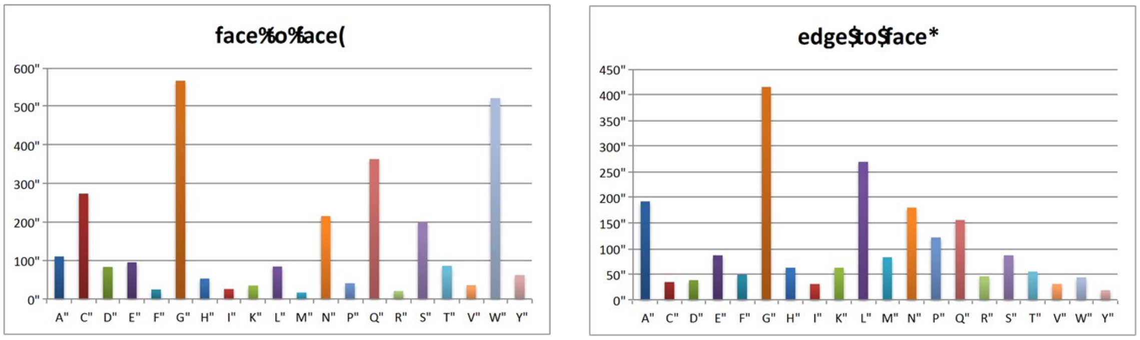

Figure S11. Distribution of the occurrence of the amide-π stacking interactions with each amino acid.

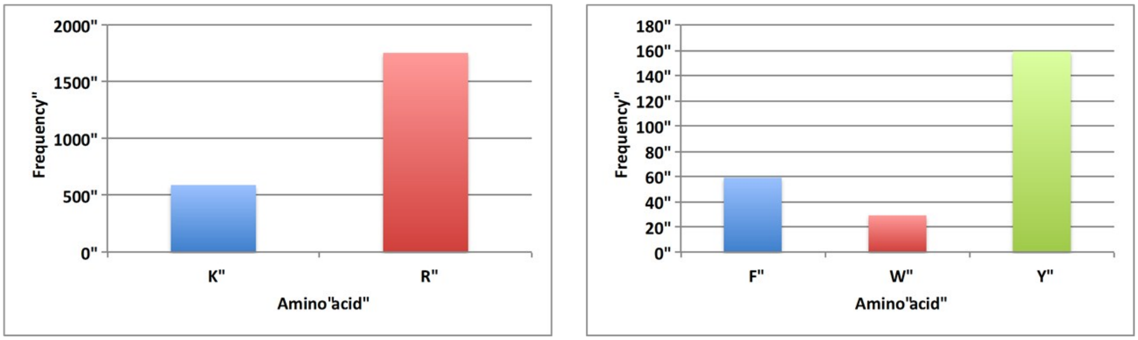

Figure S12. Distribution of the occurrence of the cation-π stacking interactions with each amino acid

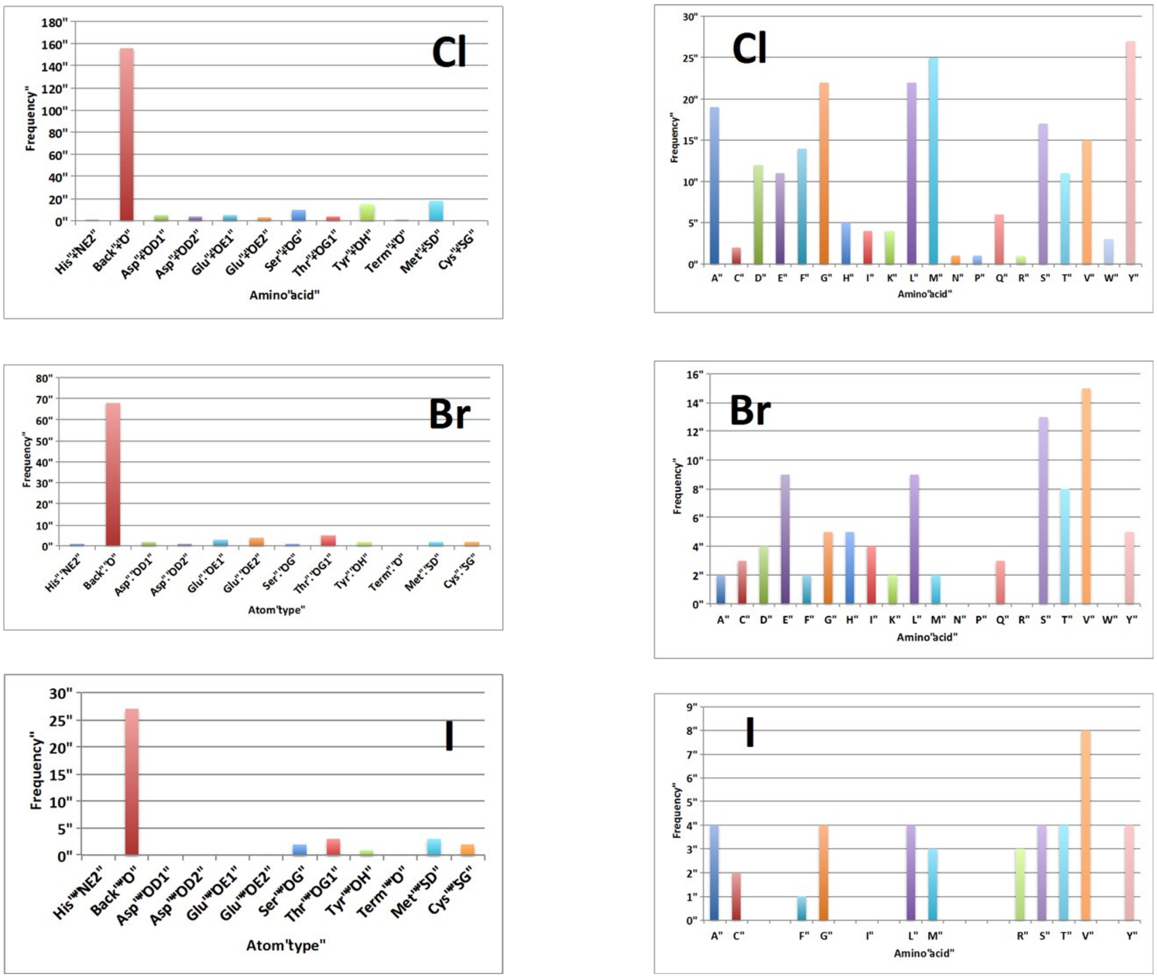

Figure S13. Distribution of the occurrence of the halogen bonding interactions with each amino acid

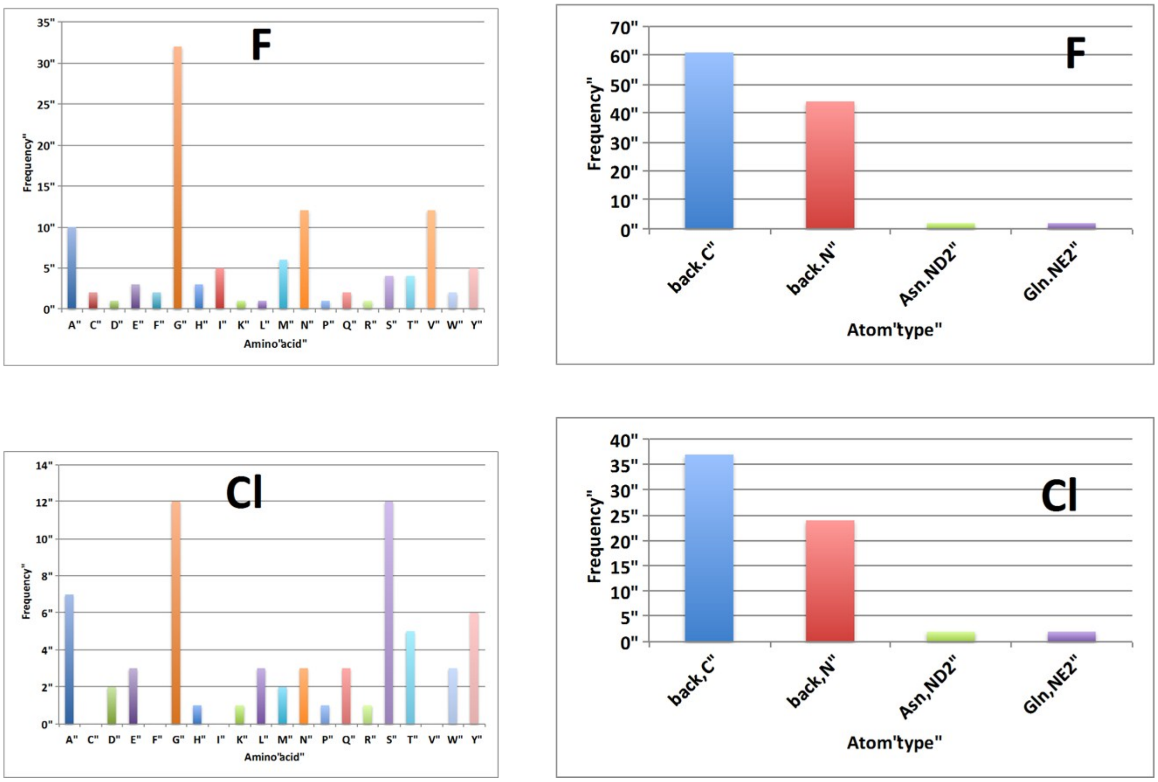

Figure S14. Distribution of the occurrence of the halogen multipolar interactions with each amino acid
